# Can Focused Ultrasound Overcome the Failure of Chemotherapy in Treating Pediatric Diffuse Intrinsic Pontine Glioma Due to a Blood–Brain Barrier Obstacle?

**DOI:** 10.3390/ph18040525

**Published:** 2025-04-03

**Authors:** Silvana Filieri, Morena Miciaccia, Domenico Armenise, Olga Maria Baldelli, Anselma Liturri, Savina Ferorelli, Anna Maria Sardanelli, Maria Grazia Perrone, Antonio Scilimati

**Affiliations:** 1 Department of Translational Biomedicine and Neuroscience, University of Bari Aldo Moro, 70124 Bari, Italy; silvana.filieri@uniba.it (S.F.); annamaria.sardanelli@uniba.it (A.M.S.); 2 Research Laboratory for the Woman and Child Health, Department of Pharmacy-Pharmaceutical Sciences, University of Bari Aldo Moro, Via E. Orabona 4, 70125 Bari, Italy; morena.miciaccia@uniba.it (M.M.); domenico.armenise1@uniba.it (D.A.); olga.baldelli@uniba.it (O.M.B.); anselma.liturri@uniba.it (A.L.); savina.ferorelli@uniba.it (S.F.)

**Keywords:** diffuse intrinsic pontine glioma (DIPG), DMG H3K27-altered, blood–brain barrier (BBB), focused ultrasound (FUS), chemotherapy, radiotherapy (RT)

## Abstract

**Background**: The blood–brain barrier (BBB) plays an important role in regulating homeostasis of the central nervous system (CNS), and it is an obstacle for molecules with a molecular weight higher than 500 Da seeking to reach it, making many drugs ineffective simply because they cannot be delivered to where they are needed. As a result, crossing the BBB remains the rate-limiting factor in brain drug delivery during the treatment of brain diseases, specifically tumors such as diffuse intrinsic pontine glioma (DIPG), a highly aggressive pediatric tumor with onset in the pons Varolii, the middle portion of the three contiguous parts of the brainstem, located above the medulla and below the midbrain. **Methods**: Currently, radiotherapy (RT) relieves DIPG symptoms but chemotherapy drugs do not lead to significant results as they do not easily cross the BBB. Focused ultrasound (FUS) and microbubbles (MBs) can temporarily open the BBB, facilitating radiotherapy and the entry of drugs into the CNS. A patient-derived xenograft DIPG model exposed to high-intensity focalized ultrasound (HIFU) or low-intensity focalized ultrasound (LIFU) combined with MBs was treated with doxorubicin, panobinostat, olaparib, ONC201 (Dordaviprone^®^) and anti-PD1. Panobinostat has also been used in children with diffuse midline glioma, a broad class of brain tumors to which DIPG belongs. **Results**: Preliminary studies were performed using FUS to temporarily open the BBB and allow a milder use of radiotherapy and facilitate the passage of drugs through the BBB. The data collected show that after opening the BBB with FUS and MBs, drug delivery to the CNS significantly improved. **Conclusions**: FUS associated with MBs appears safe and feasible and represents a new strategy to increase the uptake of drugs in the CNS and therefore enhance their effectiveness. This review reports pre-clinical and clinical studies performed to demonstrate the usefulness of FUS in patients with DIPG treated with some chemotherapy. The papers reviewed were published in PubMed until the end of 2024 and were found using a combination of the following keywords: diffuse intrinsic pontine glioma (DIPG), DIPG H3K27-altered, blood–brain barrier and BBB, focused ultrasound (FUS) and radiotherapy (RT).

## 1. Introduction

Pediatric solid tumors of the central nervous system (CNS) are the leading cause of childhood cancer death. The current treatment of brain tumors includes surgical resection whenever possible, radiotherapy and chemotherapy. The surgery is usually the first-line treatment to remove all or as much of the tumor as possible while preserving neurological functions. As for radiotherapy, there is resistance to the use of radiation in pediatric tumors because the CNS is still in development. Chemotherapy provides significant progress, but the blood–brain barrier (BBB) remains a major obstacle to be overcome to allow drugs to reach the CNS [1]. Substances that cross the BBB must have specific characteristics (Lipinsky’s rule), such as a low molecular weight and high lipophilicity, must be free or not completely bound to plasma proteins and must have stereospecificity as transport is often mediated by carriers. It is estimated that only 2% of small lipophilic molecules (molecular weight < 500 Da, LogP ≈ 2) cross the BBB and enter the CNS [2,3]. Over 98% of small molecules and nearly 100% of large molecules, such as peptides, recombinant proteins, and monoclonal antibodies, do not cross the BBB [3].

Some strategies have been developed to facilitate molecules reaching the CNS. Among these strategies, there is convection-enhanced delivery (CED), which uses a catheter that connects the target region of the brain to external sites, often combined with applied pressure that allows the delivery of the drug. A less invasive method is focused ultrasound (FUS), which has attracted interest in the treatment of various pediatric brain tumors that involves stereotaxic ultrasound.

This review reports the effect of radiotherapy, drugs and their combination on DIPG pediatric patients pre-treated with FUS.

## 2. Diffuse Midline Gliomas and Diffuse Intrinsic Pontine Glioma

According to the WHO classification, DIPG belongs to the diffuse midline gliomas (DMGs) (Table 1) [4]. They are aggressive CNS tumors affecting children and adolescents, with a median overall survival of one year from diagnosis [5]. In children, brain tumors represent 20–25% of all tumors between zero and fifteen years of age. The worldwide incidence is 2–3 cases/year/100,000 children. These tumors occur in the midline regions of the brain, specifically in the thalamus, midbrain, and pons.

**Figure 1:**
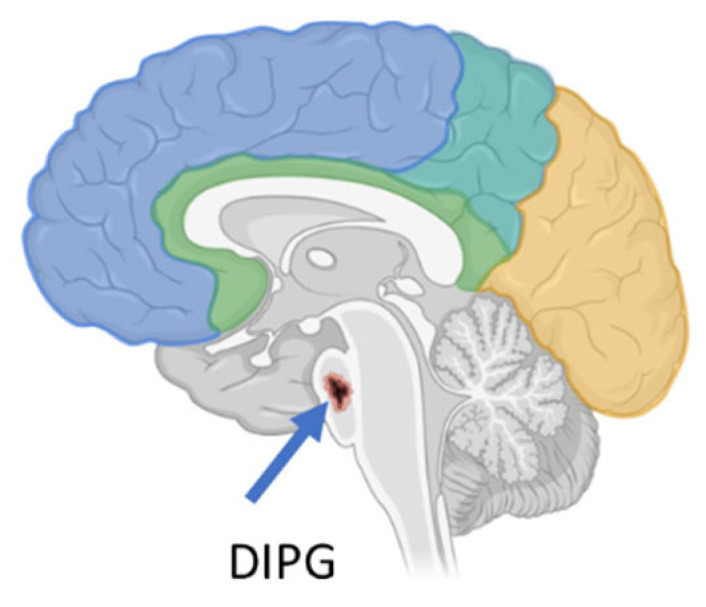
DIPG localization in the brain (created with Biorender.com).

DIPG is a high-grade glioma (h-GG) located in a critical region of the brain called the pons (Figure 1), which is responsible for controlling several vital bodily functions, such as the heartbeat, balance, swallowing, vision, and breathing. It accounts for 10% of all pediatric brain tumors. To date, DIPG remains one of the most difficult brain tumors to treat [6].

It occurs almost exclusively in children, with a mean age at diagnosis of 4–10 years. After diagnosis, the mean survival is approximately 12 months, with a life expectancy, beyond progression, of 3 months regardless of the treatment received [7,8]. Imaging is essential to establish a diagnosis of DIPG, with MRI considered the gold standard [9].

Many cytotoxic [10,11] and targeted [12,13] drugs have been studied in clinical trials for the treatment of DIPG, but unfortunately, none of these drugs have been successful, probably due to the impermeability of the BBB and the low accumulation of drugs in the tumor tissues. In brain tumors, the BBB is intact and thus prevents therapeutic agents (such as chemotherapeutics) from reaching the disease-involved area in sufficient concentrations.

Therefore, valuable delivery methods are needed to improve the efficacy of drugs directed to brain tumors.

Recent studies have identified several genetic mutations in DIPG, which contribute to the onset and development of this tumor [14]. Over 60–70% of DIPG tumors harbor heterozygous mutations at genes encoding histone H3 proteins that replace lysine 27 with methionine (H3K27M) [15].

Histones, fundamental proteins in the structuring of chromatin, are crucial for regulating gene expression. Post-translational modifications of histones, such as methylation, acetylation, phosphorylation and ubiquitination, influence DNA accessibility and, consequently, gene transcription.

Histone H3 lysine 27 is an important residue as its modifications are associated with both gene silencing and active gene transcription. In a healthy pons, H3K27 can be mono-, di- or tri-methylated (H3K27me1-me3) by the polycomb repressive complex 2 (PRC2), with the Ezh1/2 acting as the catalytic subunit [16,17]. The PRC2 complex and H3K27me3 play an important role in gene silencing during development. The DIPG-H3K27M mutation leads to an overall reduction of the trimethylation of lysine 27′s histone H3 (H3K27me3), a modification associated with the loss of gene repression and the formation of “oncohistones” that reprogram the epigenome. They, in turn, contribute to a cancer-like transcriptome and a microenvironment conducive to the proliferation of cancer cells.

## 3. Blood–Brain Barrier (BBB) Crossing

The BBB is a protective barrier for the brain. It is also a dynamic and highly selective physiological barrier for maintaining the homeostasis of the neural microenvironment by tightly regulating the bidirectional flow of neuroactive substances transferred from the blood systemic circulation to the CNS and conversely [18,19,20,21,22].

The movement of substances across the BBB occurs by various mechanisms, such as paracellular active transport, passive diffusion, solute carriers, and adsorptive or receptor-mediated transcytosis [19,21]. These transport pathways protect the CNS from both endogenous compounds and harmful xenobiotics, while simultaneously providing nutrients to CNS cells/tissues. In CNS diseases, the BBB represents a major obstacle to drug delivery, as it blocks the transport of both large and small systemically administered drugs [22]. Currently, invasive procedures are used to treat CNS diseases, which are often associated with significant risks and long recovery times.

In brain tumors, the BBB is intact and thus prevents therapeutic agents (such as chemotherapeutics) from reaching the disease-involved area in sufficient amounts to achieve sufficient concentrations and thus maximum effects. To bypass the BBB, attempts have been made to implement on-site administration of chemotherapy drugs by stereotaxic intracranial guided injection of pharmacologically active chemicals or surgically implanted reserve catheters, referred to as convention-enhanced delivery (CED), where chemotherapy reaches the tumor through a catheter under a pressure gradient to achieve greater distribution than when spread by diffusion. The catheter allows repeated administration of drugs with a small needle, and even when used for long periods, demonstrates efficacy and safety [23]. However, catheters may lead to infectious and non-infectious complications such as obstructions, misplacements and fluid loss [24,25]. In addition, neurological side effects are observed during local drug administration, such as mild to moderate headache, transient facial weakness, dysarthria, and ataxia, which disappear after stopping the infusion [26]. The understandings of all these disorders and their recovery processes can be difficult to interpret, especially in children. A standardized neurological assessment through a score is used to grade the extent of the disability of signs and symptoms of headache, ophthalmoplegia, bulbar dysfunction, paresthesia, limb weakness and cerebellar dysfunction. The score, called the “Pontine Observational Neurology Score” (PONScore), is reliable and can be used during the infusion to calculate how often and when side effects occur, and the likelihood of recovery. There are several limitations in using this method, particularly in children who already have significant pre-treatment disabilities.

It is noteworthy that temporary opening of the BBB can be achieved using focused ultrasound technology.

## 4. Focused Ultrasound’s Effect on the BBB

Focused ultrasound (FUS) is a pioneering approach to facilitating the pharmacological treatment of brain diseases. It is still an experimental technique for brain tumors, although some successes are beginning to emerge. FUS has the potential to temporarily and safely open the BBB, which is one of its most promising applications. The BBB FUS effect lasts for about 24 h [27]. This temporary opening allows better delivery of drugs directly to the tumor. Advances in the clinical applications of FUS have occurred over the last two decades, where the use of MRI and ultrasound has allowed for accurate targeting and monitoring.

FUS was first described as a method for therapeutic heating or thermal ablation of tissue [28]. The use of FUS in brain pathology began in the 1950s to obtain focal lesions in the feline brain without affecting the surrounding tissues and vasculature [29,30]. By using the correct ultrasound doses for brain neuromodulation in mammals, neuroanatomical alterations [31] are reversed [32,33].

High-intensity focused ultrasound (HIFU) and low-intensity focused ultrasound (LIFU) can be used. HIFU uses high-intensity thermal energy (>200 W/cm^2^) and LIFU uses low-intensity non-thermal energy (<100 W/cm^2^) to affect brain tissue and activity.

The HIFU wave has a narrow focus of 1 mm in diameter and 10 mm in depth to better localize the thermal effects. At the affected point, the temperature increases to more than 60 °C for several seconds, causing irreversible cell death, but without damaging the surrounding tissues where the energy density is much lower. The ability of HIFU to focus high-intensity waves allows for a very interesting use in ultrasonic surgery [34]. The optimal choice of ultrasound parameters to use depends on the depth of the target and the desired heating rate. Furthermore, FUS technology combines precision, minimal invasiveness, and the ability to reach inoperable tumors.

LIFU has a resolution at the millimeter level. It can penetrate the cerebral cortex to depths of more than 10 cm through an intact skull [35], and this suggests its use in many diseases, such as depression [36], thrombolysis in cerebral ischemia [37], Alzheimer’s disease (AD) [38] and brain tumors [39].

**Figure 2:**
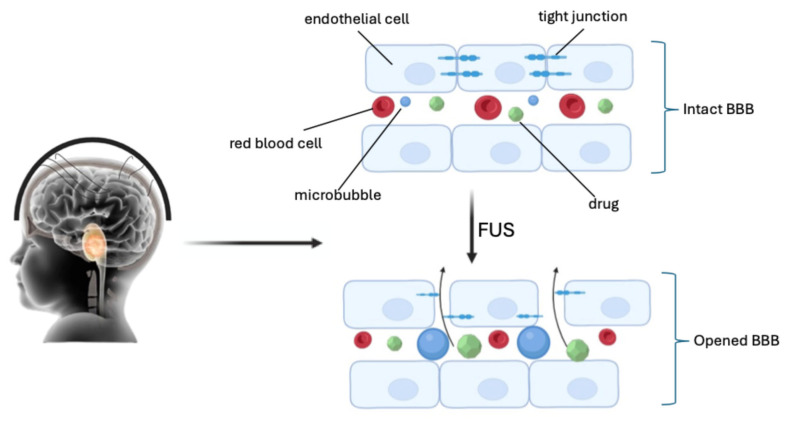
Sonoporation of the plasma membrane, disruption of the tight junctions, and opening of the BBB by FUS (created with Biorender.com).

More recent research reports the use of even low ultrasound intensities (<1 Wcm^−2^) for opening the BBB in neuro-oncology, integrated with microbubbles (FUS/MB). Microbubbles were initially developed as imaging contrast agents. They have a diameter ranging from 1 to 10 μm, with a lipid or protein coating that encapsulates high-molecular-weight gas spheres (e.g., perfluorocarbon) [40,41]. The circulating gas-filled microbubbles expand and contract under the effect of ultrasonic pressure waves (0.2–2 MHz), causing cavitation. Cavitation produces mechanical forces on the endothelium within the focal zone of the FUS, causing sonoporation of the plasma membrane, disruption of the tight junctions, and opening of the BBB (Figure 2) [42,43,44].

High-resolution 3D MRI has been integrated with the FUS system (MRgFUS) to minimize FUS’s off-target effects. MRgFUS is used to identify the focal point of the treatment site, direct the ultrasound beam, check the images in real time during treatment, and confirm the efficacy of the therapy [45,46]. In terms of the BBB, LIFU is used in a pulsed mode to reduce energy deposition [47] and prevent damage to healthy neural tissue.

Several studies have shown that the temporary susceptibility of the BBB to being crossed (BBBD) by drugs upon LIFU use is safe, without significant neuronal damage, apoptosis, ischemia or long-term vascular damage. The advantage of having microbubbles present in the blood supply is that it allows for the reduction of the ultrasound intensity, the containment of most of the disruption within the vasculature, and the reduction of the likelihood of irreversible neuronal damage. Although there are many indications that the damage can be contained to only minimal hemorrhage, the complete safety profile remains to be assessed. In addition, indications of various mechanisms, such as the dilation of vessels, temporary ischemia, mechanically induced opening of the tight junctions, and activation of various transport mechanisms, have been reported [27]. Localized BBBD can persist for a period of 3 to 24 h, depending on the intensity of the mechanical stress modulated through the ultrasound intensity and MB dose [48].

## 5. LIFU and Diffuse Intrinsic Pontine Glioma/Diffuse Midline Glioma [(DIPG)/(DMG)]

The use of FUS and FUS/MB technology to treat DIPG in association with therapeutic agents is in the initial phase. However, preliminary and interesting data are available [49].

### 5.1. FUS Combined with Radiotherapy

A promising study has been accomplished by treating B6 (Cg)-Tyrc-2J/J (B6-albino) mice with DMG cells (H3K27M-mutated) that have been intracranially implanted, as a syngeneic xenograft model of the brainstem, with FUS combined with radiotherapy (RT) to verify whether the two techniques together were safe and feasible [50]. During this study, the radiographic, physiological, and histological consequences of blood–brain barrier opening (BBBO) with FUS combined with hypofractionated RT were evaluated. In all the groups of mice, no permanent differences in the heart rate, respiratory rate, motor function, or body weight were observed, and no morbidity or mortality was observed during treatment. The BBBO was monitored by MRI, and 72 h after FUS, BBB closure was performed. However, the addition of FUS to RT did not affect survival, as all the mice had identical disease progression. Compared to the control group, both the RT only and RT plus FUS groups showed significantly slower tumor growth rates, but no significant differences were observed between the two groups. The important result observed was that in the control mice, a tumor increase was observed after 21 days, while in the treated mice, a tumor increase was observed after 44 days. These results, however, showed that hypo-fractionated RT combined with FUS was well tolerated by mice and caused the inhibition of tumor growth like that obtained in children with DIPG who were treated with conventionally fractionated RT (54 Gy) [51,52].

Recently, it has also been reported that BBBO by FUS increases the CNS-associated microglia and macrophages in the brain in healthy animals [53], while using flow cytometry, it has been shown that FUS combined with RT increases the CNS-associated microglia and macrophages in tumor-bearing animals. These encouraging results offer the possibility of associating RT and FUS in immunotherapy for the development of new treatments. Little is known about trimodal therapy involving FUS, RT and immunotherapy, and there is also a need to better characterize the tumor immune microenvironment and understand how the combination of RT and FUS can be exploited for immunotherapeutic applications [50].

### 5.2. FUS Combined with Doxorubicin

**Figure 3:**
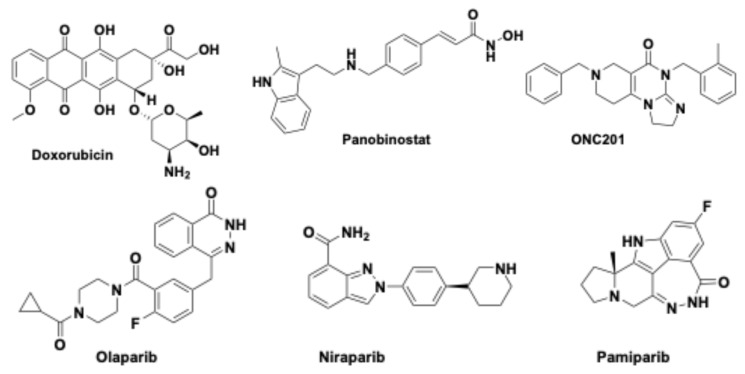
Chemical structures of doxorubicin, panobinostat, ONC201, and some PARP inhibitors (olaparib, niraparib and pamiparib).

Doxorubicin (Figure 3), a well-known chemotherapeutic agent [54], was chosen for its in vitro efficacy in relation to different DIPG cell lines.

Considering its poor BBB permeability, the preliminary study was performed on mice implanted with DIPG cells and then treated with the drug, either in the absence of FUS, in combination with FUS, or in combination with MRgFUS [55]. The findings revealed that doxorubicin had very low penetration into the CNS of the animals not treated with FUS, and low penetration in the mice treated with FUS who had doxorubicin administered intravenously. Encouraging results were obtained by administering the doxorubicin intravenously coupled with MRgFUS, which led to better opening of the BBB. After the animal was sacrificed, the brain was divided into the cerebrum, cerebellum and brainstem. The samples were then analyzed by LC-MS/MS using a triple quadrupole mass spectrometer. The level of doxorubicin detected in the brainstem was 50 times higher than that in the untreated mice. The higher amount of doxorubicin in the studied tissue remained high for two hours after administration, even considering the short plasma and tissue half-life of the free drug [55]. The consideration of having achieved a high concentration of the chemotherapeutic agent in the brainstem represents an important success, even if all this may not lead to an adequate therapeutic response in humans, considering the toxicity of the drug administered in multiple doses [56].

Other studies have been carried out on doxorubicin by inserting it into liposomes and in combination with FUS in DIPG mouse models [57]. The use of the liposomal formulation serves to reduce the toxicity as the drug is released slowly over a longer time. This study was conducted to understand how to treat pediatric DIPG, using a DIPG xenograft model. Calculating the amount of drug in the brain was quite difficult with both LC-MS/MS and high-performance liquid chromatography (HPLC), because the doxorubicin was encapsulated in liposomes. Therefore, new strategies must be implemented to determine the amount of free doxorubicin in the brain.

Overall, the FUS technique led to an increase in the concentration of the drug in the CNS but did not lead to an improvement in survival in the DIPG xenograft model, as high toxicity was observed.

Currently, two clinical trials (NCT05630209 and NCT05615623) are being conducted to evaluate the safety and efficacy of targeted blood–brain barrier disruption with an Exablate Model 4000 Type 2.0/2.1 in combination with doxorubicin treatment of pediatric patients with DIPG.

### 5.3. FUS Combined with Panobinostat

Panobinostat (Figure 3), a histone deacetylase (HDAC) inhibitor, is cytotoxic in H3K27M cell lines, both in vitro and in xenograft mouse models. It led to reduced tumor growth in H3K27M DIPG and increased H3K27 trimethylation. Although panobinostat is a small-molecule drug, once injected, it binds to the plasma albumin protein and becomes too bulky to spontaneously and effectively cross the BBB.

Low doses of panobinostat coupled with FUS/MB treatments under MRI guidance were also studied using a PDX mouse model bearing H3K27-altered DIPG and a concomitant TP53 mutation [58]. A significant increase in the panobinostat concentration was found in the parenchyma in the tumor area compared to the cortex (three-fold). The amount of panobinostat was also increased in the tumor areas treated with FUS/MB compared to those not treated with FUS/MB (over three-fold). It was demonstrated, also in this study, that FUS/MB-mediated BBB opening is accurate, precise, and reproducible.

Furthermore, during the first 4 weeks of the study, a significant decrease in tumor growth in the FUS/MB-treated mice was observed, leading to a remarkable survival benefit. An increase in the median overall survival from 21 to 31.5 days for the mice treated with FUS/MB and panobinostat compared to the mice treated with only the drug was also observed. This study can be considered an important pre-clinical investigation, preliminary to the use of this technology in clinical trials involving children and even adults with DIPG.

In fact, children with a radiological diagnosis of progressive DMG with a tumor involving the pons (intrinsic, pontine-based infiltrative lesion; hypointense in T1-weighted images (T1WIs) and hyperintense in T2 sequences, with a mass effect on the adjacent structures and occupying at least 50% of the pons) or thalami and/or histological confirmation of the H3K27M mutation of the pontine or thalamic glioma are treated with non-invasive FUS and oral panobinostat (NCT04804709). Unfortunately, the clinical trial is not yet concluded, and partial results are not available.

### 5.4. FUS Combined with Olaparib in DIPG

DMG/DIPG tumor cells are unable to repair both double-stranded DNA breaks (DSBs) due to mutations in the TP53 and defective homologous recombination (HRR) [59,60]. DSB/HRR-deficient tumors are therefore dependent on single-stranded DNA break (SSB) repair and require the enzymes poly adenosine diphosphate–ribose polymerase or poly ADP-ribose polymerase (PARP) [61]. This family of enzymes includes two isoforms, PARP1 and PARP2, which play a key role in the repair of single-stranded DNA breaks. The PARP proteins are the first to respond when DNA damage occurs. They detect it and initiate the synthesis of poly ADP-ribose (PAR) chains that act as a signal for other DNA repair enzymes. By inhibiting PARP, both PAR synthesis and DNA repair are compromised. This inhibition has been considered a therapeutic opportunity in oncology as it could enhance the efficacy of radiotherapy and of those anticancer drugs whose mechanism involves DNA damage.

Olaparib (Figure 3) is a PARP inhibitor (PARPi) with antitumor activity, already used as a first-line treatment in several tumor types [62]. Pre-treatment with olaparib, in combination with RT, inhibited cell growth and DSB repair in many cell lines in vitro, such as medulloblastoma, ependymoma, h-GG, glioblastoma and DMG [63], and in some in vivo models of lung, breast, ovarian, glioblastoma and pancreatic tumors [64,65,66].

Athymic male and female Foxn1^−/−^ nude mice were treated with olaparib combined with either radiotherapy (RT) or the FUS/MB technique in an attempt to increase the drug concentration in the brain [67]. As a result, a 5.4-fold increase in the amount of olaparib in the pons, based on the blood/tissue ratio, was found. After adding RT to FUS/MB and olaparib, a further reduction in tumor growth was observed, although not significant. In addition, these results showed significant advantages, but poor therapeutic efficacy was highlighted, as olaparib suppresses the appetite [68], reduces food intake in mice and leads to animals surviving for only 40 days after treatment due to significant weight loss. Further studies must also be carried out to evaluate the optimal time to start treatment after the inoculation of tumor cells in mice. It is necessary to consider other technical factors, such as the feasibility of continuous application of FUS/MB, to optimize the therapeutic use of FUS/MB in DMG animal models.

In addition to olaparib, other PARPi such as niraparib and pamiparib (Figure 3) have been developed, which were shown to be better BBB penetrants [69,70]. In any case, a drug that more easily permeates the BBB can invade the whole brain, losing its local specificity and probably increasing neurotoxicity. It can be concluded that the use of the FUS/MB technique has the advantage of being able to locally administer a drug that is poorly permeable.

### 5.5. ONC201 Combined with FUS

ONC201 (Dordaviprone^®^) (Figure 3) has been extensively studied in clinical trials involving children with DIPG H3K27-altered. ONC201 acts as an activator of the mitochondrial human caseinolytic protease P (*h*ClpP) [71,72,73,74,75,76,77,78]. The mitochondria, in addition to being considered the powerhouse of eukaryotic cells, also act as regulators of apoptosis, signal transduction and intracellular calcium levels [79]. The presence of chaperones and proteases within the mitochondria ensures correct functioning. Chaperones prevent and eventually correct protein misfolding, while proteases eliminate damaged proteins [80,81]. The serine protease ClpP plays an important role in proteostasis, and more recently, ClpP has been found to be involved and over-expressed in various types of cancers [82].

**Figure 4:**
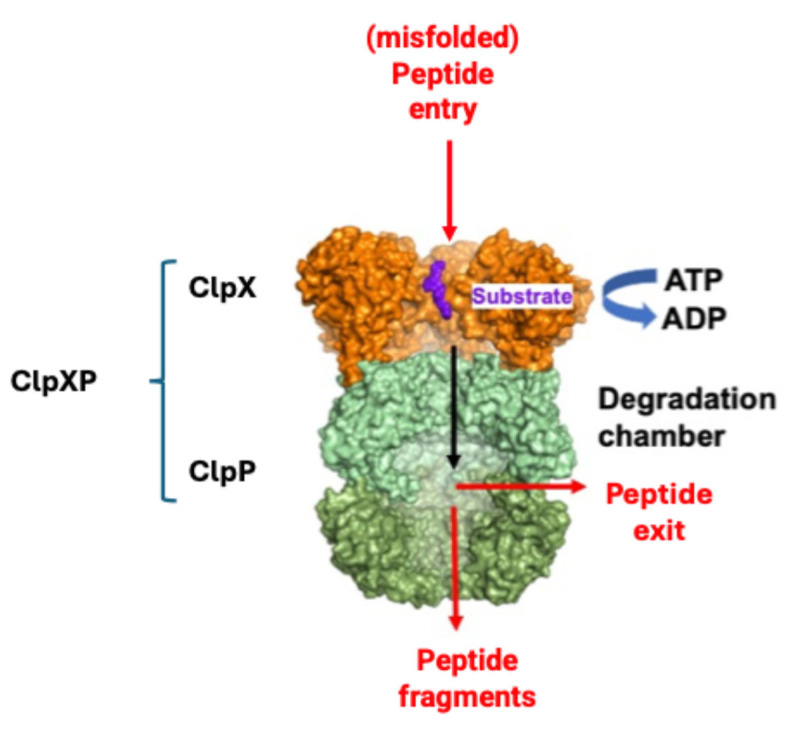
Simplified mechanism of misfolded protein identification by ClpX and degradation by ClpP (modified from [83]).

In the mitochondria, ClpP alone cannot recognize unfolded or damaged proteins to be degraded, which are in turn identified by the chaperone ClpX. When ClpP and ClpX join, they form the complex ClpXP (Figure 4), in which ClpX is formed by six identical subunits arranged to form a ring, while ClpP is instead composed of 14 subunits, organized into two heptameric rings associated with each other to form a cylinder closed at each end by the ClpX hexameric rings. The proteolytic catalytic triad Ser153, His178 and Asp227 are located inside the degradation chamber of ClpP to avoid non-specific proteolytic activities, while ClpX acts as a “gatekeeper” that selectively lets target proteins pass, favoring their degradation.

Recent research also indicates that our immune system can be important in the fight against cancer. The body’s immune response mainly involves the activation of T lymphocytes, leading to the detection and destruction of tumor cells to limit the spread of metastases. When the PD-1 receptors on T lymphocytes bind the ligands PD-L1 and PD-L2, which are expressed by tumor cells, the T lymphocytes are inactivated. In this way, tumor cells can evade the immune response.

A mouse model of syngeneic DMG was developed to study the combination therapy of FUS with ONC201, FUS with anti-PD1 and FUS with RT monitored with MRI [84].

FUS combined with ONC201 determined a decrease of the NADH-ubiquinone oxidoreductase 1 alpha subcomplex 12 (NDUFA12), an enzyme present in the mitochondrial complex 1, the largest of the five complexes of the electron transport chain (a biomarker of ONC201 response), and an increase in ROS production compared to ONC201 as monotherapy.

For the FUS and anti-PD1 combination therapy, an increase in anti-PD1 release to the brainstem was detected. The overall survival of the mice treated with the FUS and anti-PD1 combination therapy increased compared to anti-PD1 alone.

These preliminary results showed that the combination therapy of FUS and RT induced an innate immune response compared to the untreated group. The combination therapy with focused ultrasound provided a promising result by increasing the efficacy of the drugs used and of the immunotherapy.

## 6. Conclusions

In recent years, there has been a continued increase in the use of FUS in many CNS diseases to allow drugs to cross the BBB. The fundamental principles of FUS include its ability to induce thermal ablation or enhance drug delivery through transient blood–brain barrier (BBB) disruption, emphasizing the adaptability of high-intensity focused ultrasound (HIFU) and low-intensity focused ultrasound (LIFU) applications. Several ongoing clinical trials are exploring the potential of FUS in offering alternative therapeutic strategies for pathologies where conventional treatments fall short, specifically centrally located benign CNS tumors, and among those, diffuse intrinsic pontine glioma (DIPG). The aim of this review was to summarize the efforts made to prove the utility of FUS coupled with radiotherapy and/or antitumoral drugs in the treatment of CNS tumors. Articles investigating in vitro and animal xenograph models have been reviewed, adding notes on clinal trials (clinical trials.gov), trying to demonstrate the preclinical feasibility of brainstem BBB disruption using either FUS or MRgFUS. Initially, the safety and reliability of using FUS were proved, leading to studies combining various chemotherapeutic agents with FUS or MRgFUS. Initial studies were performed on orthotopic xenografts derived from patients with H3K-altered DIPG or DMG. Although great progress has been made in recent years toward understanding these diseases, few effective treatments and no cures are currently available. This is mainly due to the impermeability of the blood–brain barrier (BBB), which allows access to only 5% of the 7000 small-molecule drugs available to treat only a tiny fraction of these diseases. On the other hand, safe and localized opening of the BBB has been proven to present a significant challenge [27].

It is our hope that this review will trigger more systematic studies to definitively prove that FUS, and mainly the safer LIFU, may play an important role in bridging the BBB and providing a safe and reliable drug delivery strategy for brain diseases and for the future treatment of DIPG.

## Figures and Tables

**Table 1:** Summary of the 2021 WHO classification of CNS tumors for diffuse gliomas.

Adult Diffuse Gliomas	Pediatric Diffuse l-GG	Pediatric Diffuse h-GG
- Astrocytoma, IDH-mutant - Oligodendroglioma, IDH-mutant,1p/19q-codeleted - Glioblastoma, IDH-wildtype	- Diffuse astrocytoma, MYB- or MYBLI-altered - Angiocentric glioma - Polymorphus low-grade young neuroepithelial tumor - Diffuse l-GG, MAPK pathway-altered	- DMG, H3K27-altered - Diffuse hemispheric glioma, H3G34-mutant - Diffuse pediatric h-GG, H3-wildtype and IDH-wildtype - Infant hemispheric glioma

## Data Availability

No new data were created or analyzed in this study. Data sharing is not applicable.
